# Differences in resting state functional connectivity relative to multiple sclerosis and impaired information processing speed

**DOI:** 10.3389/fneur.2023.1250894

**Published:** 2023-10-19

**Authors:** Sean L. Carter, Ronak Patel, John D. Fisk, Chase R. Figley, Ruth Ann Marrie, Erin L. Mazerolle, Md Nasir Uddin, Kaihim Wong, Lesley A. Graff, James M. Bolton, James J. Marriott, Charles N. Bernstein, Jennifer Kornelsen

**Affiliations:** ^1^Neuroscience Research Program, Kleysen Institute for Advanced Medicine, Winnipeg Health Sciences Centre, Winnipeg, MB, Canada; ^2^Division of Diagnostic Imaging, Winnipeg Health Sciences Centre, Winnipeg, MB, Canada; ^3^Department of Clinical Health Psychology, Rady Faculty of Health Sciences, University of Manitoba, Winnipeg, MB, Canada; ^4^Nova Scotia Health and the Departments of Psychiatry, Psychology & Neuroscience, and Medicine, Dalhousie University, Halifax, NS, Canada; ^5^Department of Radiology, Rady Faculty of Health Sciences, University of Manitoba, Winnipeg, MB, Canada; ^6^Departments of Physiology and Pathophysiology, Rady Faculty of Health Sciences, University of Manitoba, Winnipeg, MB, Canada; ^7^Department of Internal Medicine, Rady Faculty of Health Sciences, University of Manitoba, Winnipeg, MB, Canada; ^8^Department of Community Health Sciences, Rady Faculty of Health Sciences, University of Manitoba, Winnipeg, MB, Canada; ^9^Department of Psychology, St. Francis Xavier University, Antigonish, NS, Canada; ^10^Department of Neurology, School of Medicine & Dentistry, University of Rochester, Rochester, NY, United States; ^11^Department of Biomedical Engineering, Hajim School of Engineering & Applied Sciences, University of Rochester, Rochester, NY, United States; ^12^Department of Psychiatry, Max Rady College of Medicine, Rady Faculty of Health Sciences, University of Manitoba, Winnipeg, MB, Canada

**Keywords:** multiple sclerosis, resting state, cognition, cognitive impairment, information processing speed, SDMT, RS-fMRI

## Abstract

**Background:**

Fifty-one percent of individuals with multiple sclerosis (MS) develop cognitive impairment (CI) in information processing speed (IPS). Although IPS scores are associated with health and well-being, neural changes that underlie IPS impairments in MS are not understood. Resting state fMRI can provide insight into brain function changes underlying impairment in persons with MS.

**Objectives:**

We aimed to assess functional connectivity (FC) differences in (i) persons with MS compared to healthy controls (HC), (ii) persons with both MS and CI (MS-CI) compared to HC, (iii) persons with MS that are cognitively preserved (MS-CP) compared to HC, (iv) MS-CI compared to MS-CP, and (v) in relation to cognition within the MS group.

**Methods:**

We included 107 participants with MS (age 49.5 ± 12.9, 82% women), and 94 controls (age 37.9 ± 15.4, 66% women). Each participant was administered the Symbol Digit Modalities Test (SDMT) and underwent a resting state fMRI scan. The MS-CI group was created by applying a *z*-score cut-off of ≤ −1.5 to locally normalized SDMT scores. The MS-CP group was created by applying a *z*-score of ≥0. Control groups (HC_MS-CI_ and HC_MS-CP_) were based on the nearest age-matched HC participants. A whole-brain ROI-to-ROI analysis was performed followed by specific contrasts and a regression analysis.

**Results:**

Individuals with MS showed FC differences compared to HC that involved the cerebellum, visual and language-associated brain regions, and the thalamus, hippocampus, and basal ganglia. The MS-CI showed FC differences compared to HC_MS-CI_ that involved the cerebellum, visual and language-associated areas, thalamus, and caudate. SDMT scores were correlated with FC between the cerebellum and lateral occipital cortex in MS. No differences were observed between the MS-CP and HC_MS-CP_ or MS-CI and MS-CP groups.

**Conclusion:**

Our findings emphasize FC changes of cerebellar, visual, and language-associated areas in persons with MS. These differences were apparent for (i) all MS participants compared to HC, (ii) MS-CI subgroup and their matched controls, and (iii) the association between FC and SDMT scores within the MS group. Our findings strongly suggest that future work that examines the associations between FC and IPS impairments in MS should focus on the involvement of these regions.

## Introduction

Multiple Sclerosis (MS) is an inflammatory and neurodegenerative disease that can cause multiple symptoms including cognitive impairment. Many individuals with MS develop impaired cognitive function, and the domain of information processing speed is affected most often in up to 51% of individuals (1). Cognitive impairment is important. In a longitudinal study of newly diagnosed individuals with MS, cognitive impairment in the form of reduced information processing speed (IPS) was associated with an increased risk of changing vocation or ceasing work (2). The neural pathophysiology underlying impaired IPS in MS is not fully understood.

Resting state functional magnetic resonance imaging (rs-fMRI) measures brain activity data while the participant is not performing a task. Analyses of the temporal correlations between activity in different brain regions provide measures of functional connectivity (FC) (3, 4). Whereas structural connectivity techniques assess the physical connection between two anatomical areas, FC speaks to the synchronicity of fluctuations in brain activity across different regions and is thought to reflect a functional relationship. A review of 86 FC studies comparing individuals with MS and healthy controls (HC) (3) reported that MS patients exhibit patterns of altered FC involving the thalamus, hippocampus, basal ganglia, amygdala, and medial temporal lobes, and reduced FC of the visual (VIS), sensorimotor (SMN), and default mode networks (DMN).

Altered FC of the thalamus, hippocampus, and temporal cortex, as well as regions associated with frontoparietal (FP), dorsal attention (DA), and DMN networks, has been linked to global cognitive impairment in MS (5). However, the literature on impaired IPS is inconsistent: the direction of FC change varies between studies (6, 7). Some previous rs-fMRI studies have found an association between IPS scores and FC measures in persons with MS (8–10) while others have not (11–15). The Symbol Digit Modalities Test (SDMT) measures IPS, and is recommended for screening of cognition in the clinical setting (1, 16). One rs-fMRI study has used a cutoff score of less than −1.5 SD below HC average on the SDMT to define a group with impaired IPS (17). The authors found increased FC for MS participants with impaired IPS as compared to those with preserved IPS. However, this study only reported global FC, and FC between specific brain regions was not described. In light of the inconsistencies across the literature to date, the association between FC and IPS in MS remains to be clearly defined.

To that end, we aimed to examine whether changes in FC could be observed using a whole-brain region-of-interest approach, comparing participants with MS and HC. In line with previous literature, we compared FC more broadly by contrasting all patients with MS to all HC, with both groups exhibiting an unrestricted range of cognitive capabilities. We hypothesized that, relative to HC, MS participants would show altered (either higher or lower) FC involving thalamus, hippocampus, basal ganglia, amygdala, and medial temporal lobes, as well as altered FC involving brain regions commonly associated with VIS, SMN, and DMN functional networks. Next, we performed three different comparisons to characterise the association between FC and IPS impairment in MS. First, we compared participants with MS and CI (MS-CI) to approximately age-matched controls (HC_MS-CI_). Since previous work that examined IPS using the SDMT has reported inconsistent results, we formed preliminary hypotheses based on previously reported FC changes in individuals with MS with global CI—we hypothesized that the MS-CI group would show altered FC in thalamus, hippocampus, temporal cortex, and regions associated with the FP, DA, and DMN. Second, to identify MS-related differences in FC that exist in the absence of cognitive impairment, we compared FC between cognitively preserved participants with MS (MS-CP) and approximately age-matched HC (HC_MS-CP_). Finally, to assess how FC relates to cognitive impairment exclusively within MS, we contrasted the MS-CI and MS-CP groups. Given that prior research has not restricted comparisons of FC specifically between cognitively preserved MS participants to cognitively preserved HC, or IPS impairment within MS, these last two contrasts were exploratory in nature.

## Methods

### Participants

Participants with MS were enrolled from a larger prospective longitudinal study of psychiatric comorbidity in immune-mediated inflammatory disorders (18). A subgroup of participants with MS were recruited for this sub-study (19) and underwent additional neuroimaging, cognitive, and psychiatric testing. Expanded Disability Status Scores (20), lesion load, and disease duration was recorded for participants in the MS group. Participants were excluded if they had comorbid neurodegenerative disease or brain tumors, or if they had any MRI contraindications. The sub-study also recruited healthy controls who did not have any chronic health conditions, history of head injury, or chronic medication use apart from oral contraceptives or hormonal replacement therapy. All participants provided written consent and were required to have sensorimotor function adequate to perform the cognitive tests, have sufficient English language knowledge to undergo the test protocol, and were aged 18 years or older.

### Cognition

Cognitive testing included the Weschler Test of Adult Reading (WTAR) (21) and the oral form of the Symbol Digit Modalities Test (SDMT) (22). The WTAR assessed premorbid intelligence (23). The SDMT is the most commonly used test of information processing speed in MS (24), and is a component of the Brief International Cognitive Assessment for Multiple Sclerosis (BICAMS) test battery (25). We used local regression-based age-, sex-, and education-adjusted SDMT scores (26) to compare the MS and HC samples. The cognitively preserved (MS-CP) group included individuals with MS whose SDMT *z*-score was ≥0 standard deviations (SD) of the HC reference group. The MS-CI group included individuals with SDMT *z*-scores <−1.5SD below the HC mean (27). The nearest age-matched participants from our HCs were selected to form control groups for the MS-CI and MS-CP groups (labeled HC_MS-CI_ and HC_MS-CP_, respectively).

### Data acquisition and analysis

The acquisition parameters and the preprocessing, denoising, and initial data analyses are described in detail in our previously published protocol paper (19). Briefly, images were collected using a 3 T Siemens TIM Trio MRI system. Anatomical T_1_-weighted images were acquired using the MPRAGE sequence. T_2_-weighted FLAIR scans were acquired for subsequent lesion detection. Resting state fMRI was acquired using the Human Connectome Project “cmrr_mbep2d_bold” sequence with an acquisition time of 7:12 min. Lesion segmentation was completed with the Lesion Segmentation Tool in SPM12, and lesion masking and filling were accomplished in FSL. Lesion-filled MS structural raw data and HC structural raw data were entered into the processing pipeline. Functional data for MS and HC were processed identically. In brief, rs-fMRI data were spatially preprocessed using SPM12 which included removal of the first 10 brain volumes, realignment, distortion correction, coregistration to MNI space, and smoothing with a 4.0 mm full-width half-maximum 3D Gaussian kernel. The images were then imported to the CONN toolbox (version 18b) (28) for subsequent pre-preprocessing. The scan-to-scan change in average BOLD signal within the global-mean mask was scaled to standard deviations and changes of >5 standard deviations were flagged as outliers which were included as a first-level covariate for scrubbing. Using the Artifact Detection Tool (29) (ART) within CONN, time points with default values of volume-to-volume intensity changes of *z* > 3.0, values of rotational motion of >0.05 degrees in any plane and/or translational motion >0.50 mm in any direction were identified. The estimated subject-motion parameters were regressed from the data in the denoising step of the preprocessing pipeline. To minimize motion-related variability in the BOLD signal, the 3 rotation and 3 translation parameters, plus their associated first-order derivatives, were regressed from the BOLD signal timeseries. The time-course of eroded white matter and cerebrospinal fluid were similarly regressed from the BOLD signal timeseries to correct for physiological noise. A temporal band-pass filter (0.008–0.09 Hz) was used to isolate only low frequency fluctuations (30).

### Analysis

We summarized participant characteristics using descriptive statistics, and compared groups using chi-square tests, Student’s *t*-tests, and Mann–Whitney U tests as appropriate.

Functional connectivity analysis was performed using brain parcellations included with CONN software (28). The CONN atlas includes 132 ROIs which are an amalgamation of the Harvard-Oxford cortical and subcortical structural atlas (31) and the cerebellar portions of the AAL atlas (32). An ROI-to-ROI analysis was used to calculate the bivariate correlations between each pair of ROIs using the General Linear Model (GLM, correlation (bivariate) settings, no weighting applied). First, we performed the broader between-subjects contrast [MS (1) HC (−1)] controlling for age and sex. Second, we performed the contrast comparing cognitively impaired MS patients with HC, controlling for sex [MS-CI (1) HC_MS-CI_ (−1)]. Third, we performed the contrast comparing cognitively preserved MS patients and HC, controlling for sex [MS-CP (1) HC_MS-CP_ (−1)]. Finally, we contrasted the cognitively impaired MS participants with cognitively preserved MS participants [MS-CI (1) MS-CP (−1)]. Significant ROI-to-ROI connections were thresholded by intensity at *p* < 0.05 corrected for multiple comparisons using the false discovery rate (33).

### Association between FC and SDMT scores in the MS group

A linear regression analysis was run for the MS group alone, identifying the association between the FC and the MS group’s SDMT scores (controlling for age). The ROI-to-ROI connection threshold was set to *p* < 0.05 corrected for multiple comparisons using the false discovery rate (33).

## Results

### Participants

We included 107 participants with MS and 94 controls after excluding 4 participants with MS and 6 HC participants due to insufficient MRI data quality. The MS group was older and had a higher proportion of women relative to the HC group. As expected, the MS group had lower SDMT scores than the HC group, but the two groups did not differ with respect to premorbid IQ based on the WTAR (Table 1).

**Table 1:** Demographic and cognitive measures for MS vs. HC.

	MS (*n* = 107)	HC (*n* = 94)	*p* (two-tailed)
RRMS *n*, (%)	89 (83.2)	–	
SPMS *n*, (%)	12 (11.2)	–	
PPMS *n*, (%)	6 (5.6)	–	
EDSS, mean (SD)	3.48 (1.5)	–	
Lesion Load, mean (SD)	11,742 (12,549)	–	
Disease Duration, mean (SD)	20.1 (12.0)	–	
Women, *n* (%)	88 (82.2)	62 (66.0)	0.015
Age, mean (SD)	49.48 ± 12.90	37.91 ± 15.43	<0.001
WTAR, mean (SD)	105.76 ± 10.86	108.87 ± 11.03	0.043
SDMT *z*-score, mean (SD)	−1.04 ± 1.21	−0.01 ± 1.01	<0.001*

Results are significant at *p* < 0.05. *indicates a one-tailed test; all others are two-tailed. RRMS, relapsing-remitting MS; SPMS, secondary progressive MS; PPMS, primary progressive MS; EDSS, expanded disability status scale; Lesion load, white matter lesion volume in mm^3^; WTAR, Weschler test of adult reading; SDMT, symbol digit modalities test.

### MS vs. HC: whole-brain ROI-to-ROI analysis

For the contrast of MS vs. HC, we found 37 pairwise differences (ROI-to-ROI pairings) that distinguished the FC of the MS group from HC (Table 2).

**Table 2:** A whole brain ROI-to-ROI analysis shows differences in functional connectivity between individuals with multiple sclerosis and healthy controls.

Seed	Target	*T*	p-FDR	r MS	r HC
MS < HC					
Putamen R	Caudate R	−5.62	0.0006	0.25	0.43
Putamen L	Caudate R	−5.17	0.0025	0.39	0.55
Hippocampus L	Vermis 10	−5.01	0.0035	−0.02	0.09
Vermis 9	ICC R	−4.82	0.0055	0.02	0.14
CO L	CO R	−4.80	0.0055	0.80	1.03
Thalamus L	Vermis 9	−4.67	0.0073	0.19	0.33
Vermis 9	ICC L	−4.66	0.0073	−0.02	0.10
FO R	FO L	−4.6	0.0080	0.41	0.60
Vermis 9	LG R	−4.46	0.0132	0.02	0.14
OP L	SCC R	−4.36	0.0162	0.22	0.39
Vermis 9	LG L	−4.34	0.0162	−0.01	0.10
Caudate R	Thalamus L	−4.22	0.0231	0.22	0.34
IFG tri R	Caudate R	−4.22	0.0231	−0.03	0.09
Thalamus R	Vermis 9	−4.17	0.0251	0.17	0.30
PaCiG L	Vermis 9	−4.11	0.0270	0.05	0.16
Accumbens L	Hippocampus R	−4.06	0.0291	0.18	0.28
OFusG L	SCC R	−4.03	0.0291	0.14	0.30
OFusG L	LG R	−4.03	0.0291	0.45	0.60
Vermis 7	SCC R	−4.02	0.0291	0.17	0.30
pPaHC R	sLOC R	−4.01	0.0291	0.12	0.24
Vermis 9	SCC L	−3.95	0.0330	−0.13	−0.03
pSMG R	Caudate R	−3.94	0.0330	−0.02	0.11
OP L	ICC R	−3.94	0.0330	0.27	0.42
Brainstem	Vermis 10	−3.84	0.0416	0.22	0.33
OP L	LG R	−3.84	0.0416	0.38	0.52
Cerebellum 3 R	Cerebellum 9 R	−3.82	0.0436	0.16	0.25
MS > HC					
IFG tri R	toITG R	4.36	0.0162	0.39	0.21
toMTG L	LG L	4.19	0.0246	0.09	−0.04
toMTG L	Cuneal L	4.13	0.0269	0.17	0.04
toMTG R	LG R	4.11	0.0270	0.08	−0.04
toMTG.L	SCC R	4.08	0.0283	0.07	−0.06
pTFusC R	SCC L	4.02	0.0291	0.32	0.20
SPL L	Cerebellum 1 L	3.96	0.0330	−0.03	−0.18
Accumbens L	Caudate L	3.90	0.0366	0.20	0.11
PT L	AC	3.89	0.0366	0.35	0.21
pMTG R	LG L	3.84	0.0418	0.16	0.04
PO L	ICC R	3.79	0.0473	0.08	−0.06

Negative *t* value indicates that FC in MS group was lower than in HC. Direction of difference and effect size is also indicated by r MS and r HC values. Results are significant after controlling for age and sex, and thresholding by ROI-to-ROI connections, FDR *p* < 0.05, two-sided. MS, multiple sclerosis; HC, healthy control; *t*, *t*-value; *p*, value of *p*; r MS, mean Fisher transformed bivariate correlation for MS group; r HC, mean Fisher transformed bivariate correlation for HC group; R, right; L, left; ICC, intracalcarine cortex; CO, central opercular cortex; FO, frontal opercular cortex; LG, lingual gyrus; OP, occipital pole; SCC, supracalcarine cortex; IFGtri, inferior frontal gyrus pars triangularis; toITG, inferior temporal gyrus temporooccipital part; toMTG, middle temporal gyrus temporooccipital part; PaCiG, paracingulate gyrus; OFusG, occipital fusiform gyrus; pTFusC, posterior temporal fusiform cortex; pPaHC, posterior parahippocampal gyrus; sLOC, superior lateral occipital cortex; SPL, superior parietal lobule; pSMG, posterior supramarginal gyrus; PT, planum temporale; AC, anterior cingulate gyrus; pMTG, posterior middle temporal gyrus; PO, parietal operculum cortex.

**Figure 1:**
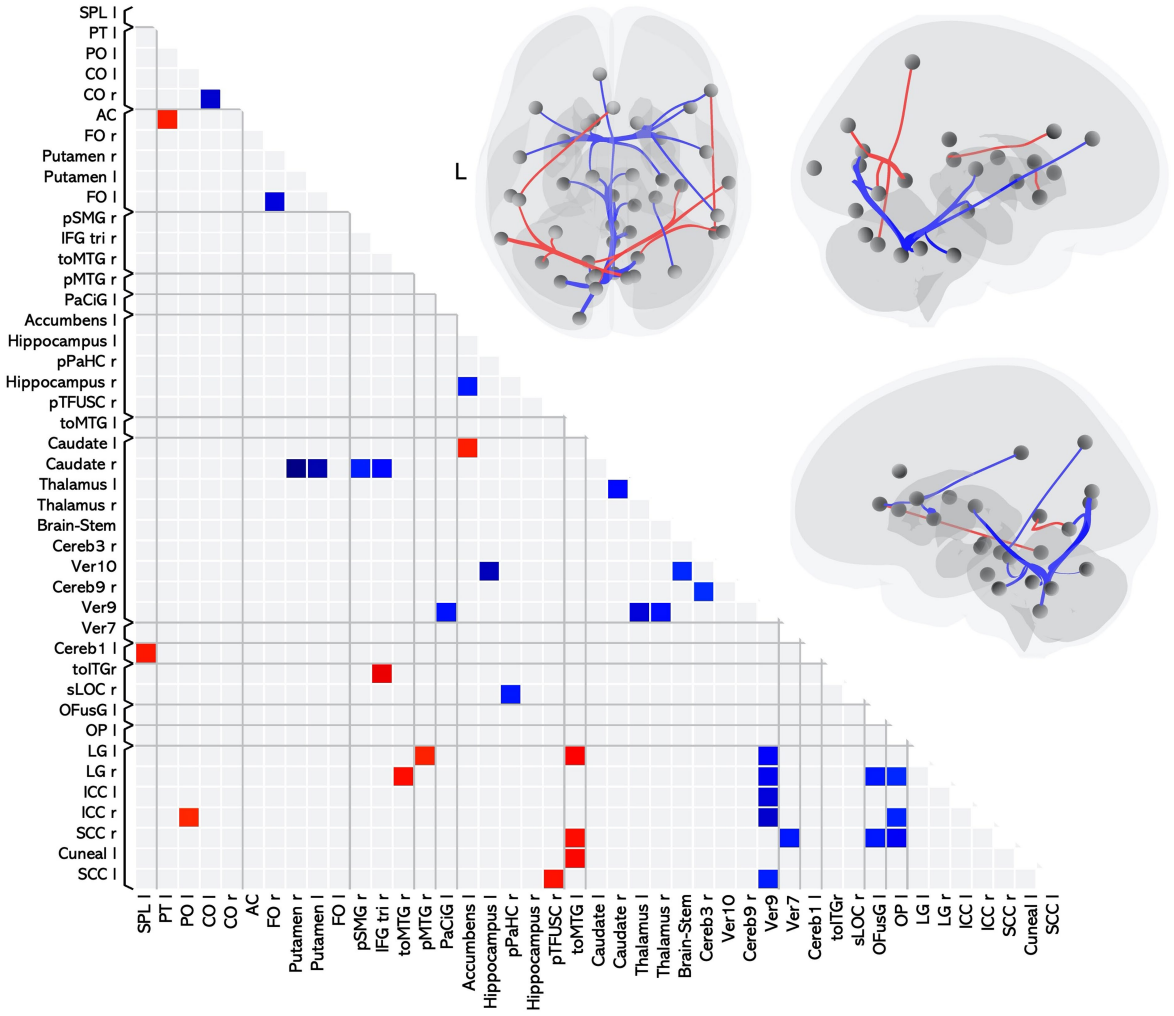
Matrix and graphic of the whole brain ROI-to-ROI contrast of MS > HC showing only statistically significant pairwise connections with increased (red) and decreased (blue) functional connectivity in the MS group as compared to the HC group. Results are statistically significant after controlling for age and sex, and thresholding by ROI-to-ROI connections, FDR *p* < 0.05, two-sided. Brain graphics are displayed in neurological convention with right corresponding to right. The first presents a transverse view looking down on the most superior aspect of the brain, with the anterior aspect towards the top of the image. The second and third are midsagittal views of the left and right hemisphere, respectively. Circles represent location of ROIs with significant effects, and connection lines represent direction of effect (red – increased FC, blue – decreased FC), not structural information. ICC, intracalcarine cortex; CO, central opercular cortex; FO, frontal opercular cortex; LG, lingual gyrus; OP, occipital pole; SCC, supracalcarine cortex; IFGtri, inferior frontal gyrus pars triangularis; toITG, inferior temporal gyrus temporooccipital part; toMTG, middle temporal gyrus temporooccipital part; PaCiG, paracingulate gyrus; OFusG, occipital fusiform gyrus; pTFusC, posterior temporal fusiform cortex; pPaHC, posterior parahippocampal gyrus; sLOC, superior lateral occipital cortex; SPL, superior parietal lobule; pSMG, posterior supramarginal gyrus; PT, planum temporale; AC, anterior cingulate gyrus; pMTG, posterior middle temporal gyrus; PO, parietal operculum cortex.

Twenty-six of these pairwise differences involved lower FC in the MS group relative to controls, shown in Figure 1 with blue squares and listed in Table 2 in the section MS < HC. Of those, 5 pairwise differences were between two ROIs associated with visual processing, and 7 pairwise differences involved at least one ROI in a brain region associated with visual processing. Two pairwise differences involved at least one ROI in a brain region associated with language processing. The remaining pairwise differences involved either a cerebellar or subcortical ROI.

Eleven of the pairwise differences involved higher FC in the MS group relative to controls, shown in Figure 1 with red squares and listed in Table 2 in the section MS > HC. Of those, 2 involved pairings between ROIs associated with visual processing, 5 involved a pairing between visual and language regions, and the remaining pairings involved other cerebellar or subcortical ROIs.

Overall, in terms of associated function, 20 of the ROI-to-ROI pairings included at least one ROI associated with visual processing, and 8 included at least one ROI associated with language. Eleven pairings included at least one ROI in the cerebellar vermis, a region associated with affective processing (34).

### Cognitive impairment

After categorization by SDMT *z*-score, we retained 43 people in the MS-CI group and 23 in the MS-CP group. Both MS-CI and MS-CP groups had a higher proportion of women than their nearest age-matched counterparts. The MS-CI group performed worse and the MS-CP group performed better on the SDMT than age-matched controls (Table 3).

**Table 3:** Demographic and cognitive measures.

	MS-CI (*n* = 43)	HC_MS-CI_ (*n* = 43)	*p* (two-tailed)	MS-CP (*n* = 23)	HC_MS-CP_ (*n* = 23)	*p* (two-tailed)
RRMS (%)	31 (77.5)	–		20 (87.0)	–	
PPMS (%)	10 (25.0)	–		0 (0)	–	
SPMS (%)	2 (5.0)	–		3 (13.0)	–	
Women, *n* (%)	33 (76.7)	27 (62.8)	*p* < 0.001	19 (82.6)	16 (69.6)	*p* < 0.001
Age, mean (SD)	52.80 ± 12.55	50.98 ± 12.31	*p* = 0.501	45.99 ± 11.41	45.35 ± 11.44	*p* = 0.85
SDMT *z*-score, mean (SD)	−2.23 ± 0.46	−0.05 ± 0.97	*p* < 0.001^*^	0.66 ± 0.63	0.11 ± 1.01	*p* = 0.031^*^

An * denotes where a Mann–Whitney U test was used in place of Student’s *t* test. Results are significant at *p* < 0.05. RRMS, relapsing-remitting MS; SPMS, secondary progressive MS; PPMS, primary progressive MS; SDMT, symbol digit modalities test.

### Cognitive impairment: whole brain ROI-to-ROI analysis

**Figure 2:**
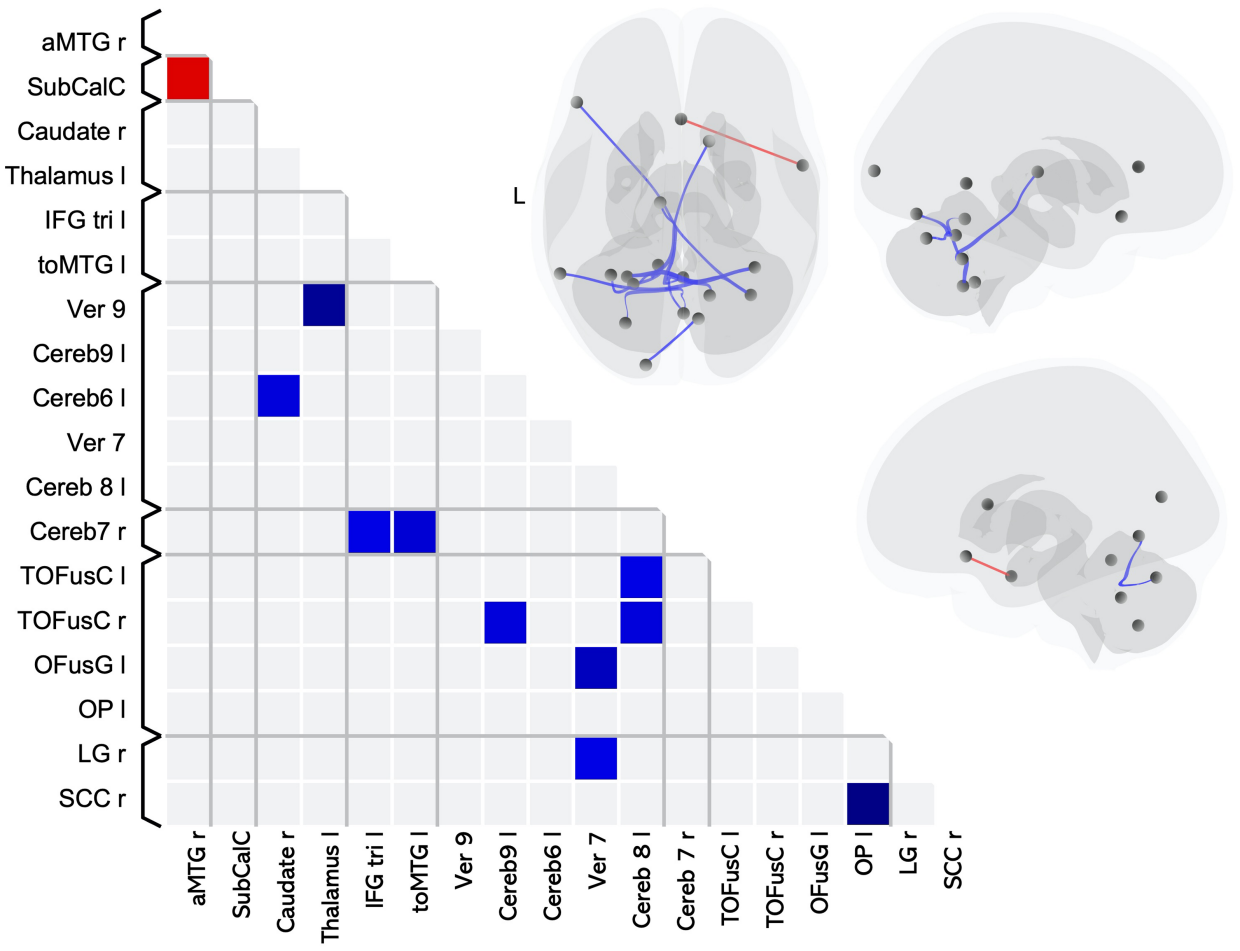
Matrix and graphic of the whole brain ROI-to-ROI contrast of MS > HC showing only significant pairwise connections with increased (red) and decreased (blue) functional connectivity in the MS-CI group as compared to the HC_MS-CI_ group. Results are significant after controlling for sex, and thresholding by ROI-to-ROI connections, FDR *p* < 0.05, two-sided. Brain graphics are displayed in neurological convention with right corresponding to right. The first presents a transverse view looking down on the most superior aspect of the brain, with the anterior aspect towards the top of the image. The second and third are midsagittal views of the left and right hemisphere, respectively. Circles represent location of ROIs with significant effects, and connection lines represent direction of effect (red – increased FC, blue – decreased FC), not structural information. MS-CI, cognitively impaired subgroup of individuals with MS; HC_MS-CI_, nearest age-matched controls for the MS-CI group; R, right; L, left; OP, occipital pole; SCC, supracalcarine cortex; OFusG, occipital fusiform gyrus; toMTG, middle temporal gyrus temporooccipital part; TOFusC, temporal occipital fusiform cortex; LG, lingual gyrus; aMTG, middle temporal gyrus anterior part; SubCalC, subcallosal cortex;IFG tri, inferior frontal gyrus pars triangularis.

We found 11 pairwise differences in FC between MS-CI and HC_MS-CI_ (Table 4), 10 of which involved lower FC for the MS group compared to the HC (Figure 2 blue squares), while one involved higher FC for the MS group (Figure 2 red square).

**Table 4:** A whole brain ROI-to-ROI analysis shows differences in functional connectivity between cognitively impaired individuals with multiple sclerosis and age-matched healthy controls.

Seed	Target	*t*	p-FDR	r MS-CI	r HC_MS-CI_
MS-CI < HC_MS-CI_					
OP L	SCC R	−5.24	0.0093	0.30	0.54
Thalamus L	Vermis 9	−5.09	0.0093	0.08	0.29
Vermis 7	OFusG L	−4.64	0.0369	0.02	0.21
toMTG L	Cerebellum 7b R	−4.4	0.0466	0.06	0.26
Cerebellum 8 L	TOFusC R	−4.39	0.0466	−0.04	0.13
Caudate R	Cerebellum 6 L	−4.36	0.0466	0.07	0.23
Cerebellum 9 L	TOFusC R	−4.34	0.0466	0.05	0.22
Vermis 7	LG R	−4.26	0.0466	0.05	0.24
IFG tri L	Cerebellum 7b R	−4.24	0.0466	0.05	0.24
Cerebellum 8 L	TOFusC L	−4.23	0.0466	0.04	0.19
MS-CI > HC_MS-CI_					
aMTG R	SubCalC	4.25	0.0466	0.29	0.14

Negative *t* value indicates that FC in MS group was lower than in HC. Direction of difference and effect size is also indicated by r MS and r HC values. Results are significant after controlling for sex and thresholding by ROI-to-ROI connections, FDR *p* < 0.05, two-sided. MS-CI, cognitively impaired subgroup of individuals with MS; HC_MS-CI_, nearest age-matched controls for the MS-CI group; *t*, *t*-value; *p*, value of *p*; r MS-CI, mean Fisher transformed bivariate correlation for MS-CI group; r HC_MS-CI_, mean Fisher transformed bivariate correlation for HC group; R, right; L, left; OP, occipital pole; SCC, supracalcarine cortex; OFusG, occipital fusiform gyrus; toMTG, middle temporal gyrus temporooccipital part; TOFusC, temporal occipital fusiform cortex; LG, lingual gyrus; aMTG, middle temporal gyrus anterior part; SubCalC, subcallosal cortex; IFG tri, inferior frontal gyrus pars triangularis.

The pairwise differences indicating lower FC for the cognitively impaired MS subgroup as compared to HC included 6 pairings that included at least one ROI in a brain region associated with visual processing and 3 pairings including an ROI in a brain region associated with language. The remaining pairwise differences involved cerebellar or subcortical ROIs.

The single pairwise difference that showed higher FC for the MS-CI group compared to their matched control group involved an ROI associated with language processing.

Overall, in terms of associated function, 6 pairings involved an ROI associated with visual processing, 3 pairings involved an ROI associated with language processing, and 6 of the pairwise differences involved a cerebellar lobule which is associated with cognition (34).

We did not find any differences in FC between the MS-CP group and matched controls (HC_MS-CP_).

We did not find any differences in FC between the MS-CI and the MS-CP groups.

### Association between FC and SDMT scores in the MS group

One pairwise connection was significantly correlated with the SDMT scores in the MS group. FC between the left cerebellum 9 and the left inferior division of the lateral occipital cortex ROIs was observed (*T* = 4.72, pFDR = 0.039).

## Discussion

We observed FC differences between MS patients and HC relative to ROIs in known visual- and language-related regions, as well as for ROIs in the cerebellar vermis. We also found widespread FC differences involving thalamic, hippocampal, and striatal areas, in line with previous studies. Similarly, in the MS-CI group compared to nearest age-matched controls, we observed altered FC in the cerebellar lobules, visual and language-related regions, as well as the thalamus and striatum. The results of our regression analysis reinforce these findings identifying one significant ROI pairing between the cerebellar 9 lobule and lateral occipital cortex related to cognition in the MS group. No differences between the MS-CP and HC_MS-CP_ groups were found, nor for the MS-CI and MS-CP groups.

Our analysis showed lower FC between cerebellar vermis 7, 9, and 10 and thalamic, hippocampal, paracingulate, and visual-related regions. The vermis is located along the midline of the cerebellum, and is usually associated with balance, posture, and coordination of eye movement (35, 36). Cerebellar involvement in MS often relates to motor dysfunction (37). However, the vermis has been implicated in affective processing across many clinical populations (34), and the vermis 9 and 10 make up a region that has been described as the “limbic cerebellum” (38). We also found lower FC between the left superior parietal lobule and cerebellar crus 1, which is located on the posterior lobe of the cerebellum and has been implicated in the cerebellar cognitive-affective syndrome (CCAS), characterized by executive function, linguistic, spatial, and affective difficulties (39, 40). Other work has implicated disruption of the cerebellar crus 1 in difficulties with language processing, mental rotation, and executive function (34). Our work reinforces recent descriptions of the role of the cerebellum in cognitive function in MS (41). Given its widespread involvement in various cognitive domains, the posterior cerebellum may be of interest in future studies of cognition in MS.

We also found FC differences in regions associated with the language network. This is one of the first to show a multitude of differences in FC in the MS group between language-related regions, and visual, caudate, and cingulate areas. There was increased FC between language-related and visual regions. For example, several visual areas (SCC, lingual gyrus) showed higher FC with the temporooccipital part of the middle temporal gyrus (toMTG) bilaterally, a region that is implicated in language comprehension and syntactic processing (42). While VIS network disruption has been noted in many studies (3), our findings emphasize increased FC between specific visual and language areas. This may be of consequence clinically; many of the cognitive measures used to assess MS, like the SDMT for IPS or other common tests for memory or executive function (43, 44), rely on visual processing and language to a large degree. It may be that increased FC between visual and language-related areas represents a network-level adaptation to structural damage, as put forward in the network collapse model of MS (45). Future studies that aim to measure the association between FC change in MS and a specific cognitive domain should account for visual or language-related cognitive difficulties in the study design.

As hypothesized based on prior findings (3), we observed altered FC in the MS group compared to controls in the thalamus, hippocampus, basal ganglia, and middle temporal lobes; the only hypothesized region which did not show differences in FC was the amygdala. However, our findings typify the literature: we identified significant differences in FC at commonly cited ROIs. In addition, we found different patterns of FC. For example, previous work on the thalamus has described increased FC with motor, occipital, temporal, and subcortical areas. By contrast, we found lower FC between the bilateral thalamus and cerebellar vermis 9, and lower FC between the left thalamus and right caudate. So too do we find this with the hippocampus: where some authors report the opposite (3), we found lower FC between the hippocampus and the nucleus accumbens. Our findings in the basal ganglia agree with the more detailed FC analysis of the striatum conducted by Cui and coauthors (46). We identified lower FC between the right caudate and putamen. However, our analysis differs from theirs in that we showed increased FC between the right caudate and the inferior frontal gyrus and they did not, while they reported many alterations in FC in the MS group that we do not.

We found widespread altered FC in brain regions associated with the VIS network that are consistent with previous work, though the direction of FC differences varies (3). These included V1 (intra- and supracalcarine cortex), the occipital pole, fusiform cortex, and lingual gyri. In contrast with previous work, we did not see altered FC in any regions associated with the DMN or SMN. While this is surprising given the number of studies which report on these networks, no consensus on the nature and direction of altered FC of the DMN or SMN in MS has been reached (6, 7).

Based on previous studies, we did not expect to see changes in FC at regions other than those that are associated with the VIS, DMN, or SMN. However, we found increased FC in the MS group between the left planum temporale, a region involved in complex auditory and speech processing (47, 48) and the anterior cingulate gyrus (AC), a region involved in assessing the reward value of actions (49). The AC is considered to be part of the salience network (SN), which functions to orient attention towards relevant stimuli. Within the SN, the AC is particularly responsible for generating motor, cognitive, and behavioural responses to those stimuli (50). That the SN is implicated in MS-related cognitive dysfunction agrees with a recent graph theory study (9), but their results do not name the AC specifically.

### Cognitive impairment

In the group of MS-CI individuals with impaired IPS, we showed altered FC in the cerebellum, thalamus, and basal ganglia, as well as areas associated with VIS and LAN networks. Current literature on associations between FC and IPS is contradictory, with conflicting findings on the correlation between SDMT scores and FC measures (1, 7, 10, 14, 15). Using a definition of the MS-CI group that is similar to our own (26, 27), Meijer et al. (17) examined the difference between CI and CP groups in whole-brain FC and structure. They reported that, alongside worsened structural integrity and reduced deep brain grey matter volume, their CI group also showed an increased FC metric compared to CP individuals with MS. Given the differences in study methodology and that the authors did not include the cerebellum in their analysis, only limited comparisons are possible. We found mainly lowered FC between cerebellar lobules and visual ROIs in our MS-CI group, as well as increased FC between right aMTG and the subcallosal cortex. More work that explores specific brain regions in an MS-CI group is needed.

There were FC changes in common at 2 ROI pairs between the analysis of all MS and HC, and the analysis of our MS-CI group compared to nearest age-matched controls. Both the MS group and the MS-CI group had lower FC between left occipital pole and right SCC and lower FC between left thalamus and cerebellar vermis 9. These results are consistent with previous systematic reviews which indicate altered FC involving thalamic and visual areas in MS (3, 6). They also reinforce the results of the first analysis we performed by indicating the same cerebellar, thalamic, and visual ROIs. In contrast, we also found altered FC between 9 ROI pairs in the MS-CI group that were not implicated, pairwise, by the analysis of the entire MS group. It is notable that 8 of the 9 ROI pairs involved lower FC of cerebellar ROIs with subcortical, visual, and language-related regions. Similarly, the connection between the left cerebellar 9 lobule and the inferior lateral occipital cortex being related to SDMT scores in the MS group complements the finding from the group-wise comparisons highlighting the relevance of FC between cerebellar and visual cortex to impaired cognition in MS.

We propose that regions of FC change in common between MS versus HC and MS-CI versus HC_MS-CI_ (i.e., thalamus, cerebellar vermis 7 and 9, OP, SCC) may represent the FC changes that are common to individuals with MS. A larger sample size of cognitively preserved individuals with MS may then detect changes in these same common regions. The additional regions present only in the MS-CI vs. HC_MS-CI_ contrast (i.e., cerebellum 6, 7b, 8) may represent a signature of additional IPS impairment. FC of cerebellar lobule 9 was implicated in impaired cognition in MS, based on the results of our linear regression of SDMT scores in the MS group. These posterior cerebellar lobules should be used as seeds in follow-up analyses that examine the relationship between FC and IPS in individuals with MS. While we observed ROI pairings that significantly differed between cognitively impaired participants with MS and age-matched healthy controls, these results were less robust than those observed in the overall MS vs. HC contrast. We speculate that this may be due to a combination of the fact that (1) the contrast between cognitively impaired participants with MS and healthy controls provides a narrower reflection of differences between MS and healthy state, relative to the FC underlying information processing speed impairment and (2) the contrast between cognitively impaired MS participants and their matched healthy controls included a smaller sample size for the contrast groups, compared to the overall MS vs. HC contrast. Thus, this contrast had less power to detect group differences than the overall MS vs. HC contrast. With a larger sample size of cognitively impaired participants with MS and controls, it may be possible to detect additional differences. In addition, larger sample sizes of cognitively impaired MS participants would support analyses focused on individual variability and associations between MS, FC, and cognitive impairment. Future work should attempt to replicate these findings in larger, more diverse samples, and should investigate the network properties of these regions. We expect that this will bring further insight into the FC changes that underlie MS-related declines in IPS.

The contrast between HC and participants with MS and CI cannot distinguish FC differences related exclusively to CI from those related to MS, given that the groups varied both in their cognitive status as well as by MS status. This was the rationale for including the contrast of the cognitively preserved MS participants and their matched healthy controls, and the contrast of the cognitively intact vs. cognitively preserved participants within the MS group. Our results did not isolate FC differences specific to MS without cognitive impairment in our comparison of cognitively preserved MS patients to HC. Neither did we isolate FC differences specific to cognition impairment in MS in our comparison of cognitively impaired vs. cognitively preserved individuals with MS. The small sample size of the MS-CP group, which was common to both contrasts, may account for this. Future work using a larger sample size of cognitively preserved participants with MS will determine if this is a false negative due to low power.

### Limitations and future directions

We made attempts to follow the standardization and study quality recommendations from Jandric and coauthors when designing this study, which include the study of phenotype-specific influences, controlling for age, using well-established measures, defining ROIs consistently with previous research, and conducting model-led research (7). We controlled for age as a covariate of no-interest in our analysis or by age-matching. We used an established and validated cognitive measure and a standard brain atlas for parcellation. We fell short of the recommendations in two ways. The number of MS participants with primary progressive MS and secondary progressive MS was small and we were therefore unable to consider potential differences by MS subtype. However, many studies to date have used either mixed samples or a sample consisting of solely individuals with relapsing-remitting MS (7). Our samples were appropriate to our aim of describing broad trends in FC that apply across disease subtypes. Finally, we did not design our study to assess a specific disease model; however, we expect that the results obtained here will inform hypotheses for future model-led study designs. For example, much work remains to determine the adaptive vs. maladaptive implications of increases and/or decreases of FC in MS compared to healthy state. Many studies have now identified difference in FC for those with MS compared to the healthy population (3), however, far fewer studies have examined how resting state FC relates to cognitive impairment in MS (9–16, 18). Without longitudinal research to allow assessment of changes over time, in association with changes in cognition, it is challenging to assert whether the differences in FC that we have observed in the present study reflect adaptive (compensatory) vs. maladaptive changes in those with MS. Continued research in this area is necessary to fully elucidate the implications for the difference in FC presently observed. Further, the objective of the present study was to identify differences between those with MS and healthy controls relative to the FC underlying cognitive impairment. Informed by the present findings, future research can and should aim to determine potential causal mechanisms underlying the differences in cognitive-impairment related-FC between those with MS and HC. This could include, for example, exploring how individual variability in measures like lesion load, which are central to the pathology of MS, relate to cognitive impairment and associated differences in FC.

## Conclusion

Using a whole-brain ROI-to-ROI approach, we found significant differences for FC among those with MS compared to healthy controls, with ROI-pairings involving the affective and cognitive regions of the cerebellum, as well as visual and language-associated regions. Our results were generally concordant with recent findings demonstrating altered FC in subcortical and visual structures in persons with MS, but the precise direction of FC changes was not always consistent. In contrast with previous work, we did not show altered FC at regions associated with the DMN or SMN. We report that individuals with MS and impaired IPS have altered FC of the cerebellum, visual-related, and language-related areas. The regions we identified on the posterior lobe of the cerebellum have not been linked to impairments in IPS in MS previously. We suggest that further work examines the associations between IPS and FC of these regions in persons with MS.

## Data availability statement

The datasets presented in this article are not readily available because some participants did not agree to data sharing. Components of the datasets may be made accessible to qualified investigators with the appropriate ethical approvals and data use agreements upon request. Requests to access the datasets should be directed to RM, rmarrie@hsc.mb.ca.

## Ethics statement

The studies involving humans were approved by University of Manitoba Bannatyne Research Ethics Board. The studies were conducted in accordance with the local legislation and institutional requirements. The participants provided their written informed consent to participate in this study.

## Author contributions

SC, JK, RM, JF, and RP contributed to the study design. SC, RP, JF, CF, RM, EM, MU, KW, LG, JB, JM, CB, and JK participated in data acquisition, processing, revised the manuscript, and approved the final version. SC and JK conducted the analyses and drafted the manuscript. All authors contributed to the article and approved the submitted version.

## Group members of the Comorbidity and Cognition in Multiple Sclerosis (CCOMS) Study Group

Ruth Ann Marrie, Charles N. Bernstein, Jennifer Kornelsen, John D. Fisk, Ronak Patel, Chase R. Figley, Md Nasir Uddin, Sean L. Carter, Kaihim Wong, Teresa D. Figley, Carl A. Helmick, Erin L. Mazerolle, Christopher B. O’Grady, Salina Pirzada, Marco R. Essig, Lesley A. Graff, James M. Bolton, and James J. Marriott.
